# COVID-19 Severity in Patients With Apical Periodontitis: A Case Control Study

**DOI:** 10.1016/j.identj.2024.01.002

**Published:** 2024-01-20

**Authors:** Nadya Marouf, Raidan Ba-Hattab, Fatima Al-Sheeb, Amal Diab, Hanan Diab, Maryam Al-Majed, Khalid Al-Haithami, Ghanim Al-Mannai, Noha Barhom, Shailaja Tharupeedikayil, Faleh Tamimi

**Affiliations:** a Division of Endodontics, Department of Dentistry, Oral Health Institute, Hamad Medical Corporation, Doha, Qatar; b College of Dental Medicine, QU Health, Qatar University, Doha, Qatar; c Hamad Dental Center, Hamad Medical Corporation, Doha, Qatar

**Keywords:** Biomarkers, Inflammation, Oral diseases, Periapical diseases, Radiography panoramic, SARS-CoV-2

## Abstract

**Objectives:**

Apical periodontitis (AP) has been associated with systemic inflammatory biomarkers that have also been associated with COVID-19 severity. This study was designed to test the hypothesis that the presence of apical periodontitis could be associated with increased risk of COVID-19 complications.

**Methods:**

A case control study (*N* = 949) was performed using the medical and dental records of patients diagnosed with COVID-19 in the State of Qatar between March 2020 and February 2021. Cases comprised COVID-19 patients (*n* = 63) who experienced complications (death, intensive care unit admissions, mechanical ventilation), and controls were COVID-19 patients (*n* = 886) who recovered without such complications. The presence of periapical apical periodontitis was assessed on the radiographic records taken prior to COVID-19 infection. Associations between apical periodontitis and COVID 19 complications were analysed using logistic regression models adjusted for demographic and medical factors. Blood biomarkers were assessed in both groups and compared using the Kruskal–Wallis test.

**Results:**

COVID-19 complications were found to be associated with the presence of apical periodontitis (adjusted odds ratio = 2.72; 95% CI, 1.30–5.68; *P* = .008). Blood analyses revealed that COVID-19 patients with apical periodontitis had higher levels of white blood cells and haemoglobin A1c than the patients without apical periodontitis.

**Conclusions:**

The presence of apical periodontitis could be associated with increased risk of COVID-19 complications.

## Introduction

COVID-19 caused by the SARS-CoV-2 virus has presented a great challenge.^1^ Whilst most COVID-19 patients do not experience serious consequences from the infection, a small percentage of patients can face severe complications including death. Severe COVID-19 has been associated with comorbidities (ie, obesity, diabetes, advanced age)^2^ and increased serum levels of inflammatory biomarkers (ie, C-reactive protein [CRP]).^3^^,^^4^ Oral diseases are also associated with systemic inflammation; hence the reason why several studies have investigated the possible associations between oral diseases and COVID-19 severity. For instance, poor oral health was found to be correlated with increased CRP levels in COVID-19 patients,^5^ and retrospective studies have found an association between periodontitis and COVID-19 severity.^6^, ^7^, ^8^

It has been hypothesised that these possible associations between chronic oral inflammatory conditions such as periodontitis and COVID-19 complications could stem from a priming effect of oral infections on systemic inflammation.^9^ Studies have shown that patients with underlying inflammatory conditions are more susceptible to severe COVID-19. In these patients, increased levels of inflammatory biomarkers prior to SARS-CoV-2 infection would increase even further after infection. This phenomenon has been observed in patients with periodontal disease whose increased serum levels of inflammatory biomarkers seem to make them more susceptible to abnormally higher inflammatory biomarker levels and more severe COVID-19 when infected with SARS-CoV2.^10^ These observations have led researchers to question whether other inflammatory conditions of the oral cavity, such as apical periodontitis (AP), could also be associated with COVID-19 complications.

Apical periodontitis (AP) is a common chronic inflammatory condition caused mostly by caries, leaking restorations, and trauma, resulting in progressive bacterial colonisation of the dentin–pulpal complex and eventually the periapical tissues, causing a characteristic bone resorption.^11^ It is diagnosed radiographically by the presence of a radiolucent area around the roots, with breakage of the periodontal ligament (PDL).^12^ Most apical radiolucencies (75%) are related to AP,^13^ and around 30% of the global population may have at least 1 AP lesion; this could be even higher in certain populations.^14^, ^15^, ^16^

AP infections can trigger a systemic inflammatory response that has been associated with increased oxidative stress and high blood levels of inflammatory biomarkers such as C-reactive protein (CRP), interleukin (IL)-1, IL-2, and IL-6.^17^, ^18^, ^19^, ^20^, ^21^, ^22^ In severe cases, this inflammatory response may even evolve into septicaemia and increase the risk of pathologic conditions such as atherosclerosis,^23^ cardiovascular disease, and diabetes.^24^ Since the abovementioned biomarkers and conditions have also been linked to increased risk of severe COVID-19, AP could also be associated with severe COVID-19 outcomes. Accordingly, the aim of this case control study was to test this hypothesis. A secondary aim of this study was to investigate the association between AP and inflammatory biomarkers of COVID-19.

AP shares aetiologic factors with periodontitis (ie, poor oral hygiene and anaerobic infections).^25^^,^^26^ The Gram-negative anaerobes native to the infected root canal system can also be involved in periodontitis,^20^ and necrotic pulp tissue can become coinfected with periodontal pathogens through the lateral canals and denuded dentine surfaces.^27^ Moreover, both periodontitis and AP contribute to low-grade systemic inflammation and increased serum levels of inflammatory biomarkers associated with severe COVID-19 (ie, CRP, IL-1, IL-2, IL-6, and C3).^22^^,^^26^ However, there are some differences between AP and periodontitis; each one of this conditions is mainly dominated by different species of pathogens,^20^^,^^27^ and there are also differences in the inflammatory signature of both conditions. AP is characterised by increased serum levels of immunoglobulin (Ig)A, IgG, and IgM,^17^ whilst periodontitis is characterised by increased levels of ferritin and d-dimer.^28^ Given the links between AP and periodontitis, in this study we also investigated possible synergies between these 2 conditions in COVID-19 patients.

## Methodology

### Study population

This retrospective study was conducted amongst COVID-19 patients in the state of Qatar using the national electronic health records hosted at the Business Intelligence Unit of Hamad Medical Corporation (HMC). In Qatar, all COVID-19 patients are solely managed by the government health care sector, which also provides medical and oral health care services to a large segment of the population of the country, and it has a unified electronic health record system (Cerner), which includes dental and medical records. Ethical approval for this retrospective case control study was obtained from the Institutional Review Board of HMC with a waiver of informed consent that was granted due to the retrospective nature of data retrieval (protocol number MRC-01–20–1228).

In this study, we included adult patients (≥18 years) diagnosed with COVID-19 during the period between February 28, 2020, and December 31, 2020, who recovered or died from the disease before February 28, 2021. In order to be included in the study, the patients also had to have active dental records with at least 1 digital orthopantomogram (OPG) before COVID-19 infection, during the period between March 1, 2019, and December 30, 2020. Exclusion criteria were the following: edentulous, with poor quality or poorly taken OPGs, patients with active orthodontic treatment, and if OPGs were taken after COVID-19 diagnosis or before March 2019. This observational study was reported following STROBE checklist.

### Study design

In this case control study, cases were defined as COVID-19 patients with severe complications such as death, admission to the intensive care unit (ICU), or management with assisted ventilation, whilst controls were defined as those who recovered without any of the abovementioned complications. All controls were included for analysis and no matching was performed. The exposure variable was the presence of AP.

### Data extraction

All medical data from cases and controls, including COVID-19 outcomes, predisposing conditions, and blood result analyses were extracted from the electronic records of HMC as described previously.^7^ Known potential confounders such as sex, age, and citizenship status (as a marker for socioeconomic status), dental appointment track record (as a marker of oral health attitudes), smoking habits, cardiovascular diseases, diabetes mellitus, hypertension, chronic obstructive pulmonary disease, chronic kidney and chronic liver diseases, organ transplant, cancer, chemotherapy, and autoimmune diseases were considered present when at least 1 related ICD-10 code was found in the patient's record prior to COVID-19 infection. Information on the number of dental appointments (appointments given, broken appointments, cancellations, and appointments attended) were registered prior to COVID-19 between 2018 and 2020. Body mass index (BMI) was categorised as overweight/obese (BMI ≥ 25 kg/m^2^) and adequate/underweight (BMI < 25 kg/m^2^); smoking was categorised as current/past and never; and diabetes was categorised as present or absent. For the other chronic conditions, we created a variable “comorbidity” by computing the presence of each of the above conditions. Values were categorised according to the number of comorbidities into 0, 1, and ≥2.

Laboratory data on biomarkers measured on patient admission (initial) and before patient discharge (latest) were extracted from the records. This included white blood cells (WBC; 10^3^/µL), lymphocytes (10^3^/µL), IL-6 (pg/mL), CRP (mg/L), d-dimer (mg/L), haemoglobin A1c (HbA1c; %), creatinine (µmol/L), urea (mmol/L), vitamin D (ng/mL), and ferritin (µg/L).

### X-ray assessments

The OPG radiographs of the included patients were identified in the database and assessed using the software XELIS Dental 1.0 and INFINITT Dental PACS (INFINITT Healthcare Co. Ltd.). Images were adjusted for contrast for each tooth as needed to trace the periodontal ligament space. X-ray assessments were done by a team of 8 examiners with more than 10 years of experience in endodontics (NM, FS, AD, HD, MM, KH, RH, ST). Prior to analysing the OPGs included in the study, all the examiners were calibrated by a senior endodontist (GM) on diagnosing the presence of radiographic AP through the 3-step calibration exercise described below. First, a training session on the periapical index suggested by Orstavik et al^29^ was provided to explain to the reviewers the guidelines for AP diagnosis.^29^ Then each reviewer was given 20 OPGs for independent analysis. Discrepancies in diagnosis were discussed in a follow-up session. The exercise was repeated until a kappa index of 85% was reached, which was achieved after 2 rounds of assessments.

Each of the x-rays included for assessment was assessed by at least 2 independent examiners blinded to the medical data and to each other's assessment. Disagreement between the examiners were settled by a third examiner (GM) blinded to the observations of the original examiners and to the medical data. The majority diagnosis was considered.

A given tooth or residual root was considered to have an AP lesion if the apical periodontal ligament space was at least twice as wide as at the lateral PDL space at midroot (Peri-apical index score of ≥3)^30^ and it was in continuation with the PDL. If an AP lesion was not detected, the tooth was considered healthy (H). Root canal fillings were defined as radiopaque material in the canal space or pulp chamber (RCT). AP in root-treated teeth was marked as secondary apical periodontitis (SAP), whilst AP lesions in nontreated teeth were registered as primary apical periodontitis (PAP). Root-filled teeth without AP were marked as healthy root canal–treated teeth (HRCT). Teeth were considered unsuitable for diagnosis (not applicable [NA]) if they were impacted or overlapped by adjacent anatomic structures, if they had immature or resorbed apices, and if 2 examiners were unable to trace the PDL.

Root canal treatments or re-treatments completed before COVID-19 infection were considered HRCT. Teeth absent on the OPG or extracted before COVID-19 diagnosis were recorded as missing (X). Teeth with radiographic signs of AP that underwent extraction or complete root canal treatment or re-treatment before COVID-19 infection were not considered with AP because although the treatment outcomes could not be evaluated, it could be assumed that the AP was healed given the fact that treatments were done by experienced endodontists following robust clinical guidelines. The OPGs were also assessed for the presence of periodontitis using the new 2017 classification as described in another study.^7^

## Sample size calculations and statistical analysis

Sample size was calculated using the g-power software based on the observations of previous studies on oral health and COVID-19.^7^^,^^10^ We calculated that in order to obtain 85% power (1-β error probability) with an α error of 0.05, an effect size of 0.02, and 10 predictors, an estimated sample of 914 was deemed necessary. Associations between AP and COVID-19 complications were analysed using logistic regression because this statistical method is able to assess the magnitude of the association between an exposure and a specific disease whilst adjusting for potential confounders. In the logistic regression model, patients were categorised as those with AP (≥1 lesion[s]) or without AP, and the model was adjusted for potential confounders known to be associated with COVID-19 severity and oral health such as demographic, medical, and behavioural factors. More specifically, the model was adjusted for age (as a continuous variable), sex, periodontitis, citizenship, smoking, hypertension, BMI, diabetes, attended dental appointments per year, and number of other comorbidities. The variable “number of other comorbidities” included any of the following comorbidities: asthma, autoimmune diseases, cardiovascular diseases, chronic obstructive pulmonary diseases, cancer, chronic kidney diseases, chronic liver diseases, history of organ transplantation, or immunocompromising conditions. These medical conditions were selected for our model because they have been found to be associated with COVID-19 complications.

In order to adjust for socioeconomic status, health care system access, and oral hygiene practices, the logistic regression model was adjusted for citizenship and dental appointment trajectory. These parameters are strong indicators of socioeconomic status and access to health care and oral hygiene practices, respectively. In Qatar, patients’ citizenship is a very strong predictor of socioeconomic status, as it is highly correlated to income and access to care. Qatari citizens have an annual income that is significantly higher than noncitizens and also have preferential access to the health care system and disease prevention programmes. The number of dental appointments attended by the patients between 2018 and 2020 was used to account for oral hygiene practices, as dental visiting trajectory has been associated with oral health practices as well as with socioeconomic status.^29^

The number of teeth per patient labeled as PAP, SAP, X, HRCT, H, and NA were compared between patients with COVID-19 complications and those without. Also, blood parameters on initial COVID-19 diagnosis and prior to death or discharge were compared using the Kruskal–Wallis test. All statistical analyses were done using SPSS, version 20.0 (IBM Inc.). Statistical significance was set at a *P* value <.05. In addition, a subgroup analysis was performed in patients with or without periodontitis to differentiate the effect of the 2 main types of oral infections on COVID-19 complications.

## Results

Out of the 141,422 COVID-19 cases registered in Qatar by December 31, 2020, 2621 had digital dental records in the system. Amongst these, 1672 were exclude based on our inclusion and exclusion criteria and 949 patients were included for analysis. No patient was vaccinated by the time of SARS-CoV-2 infection.

**Fig. 1:**
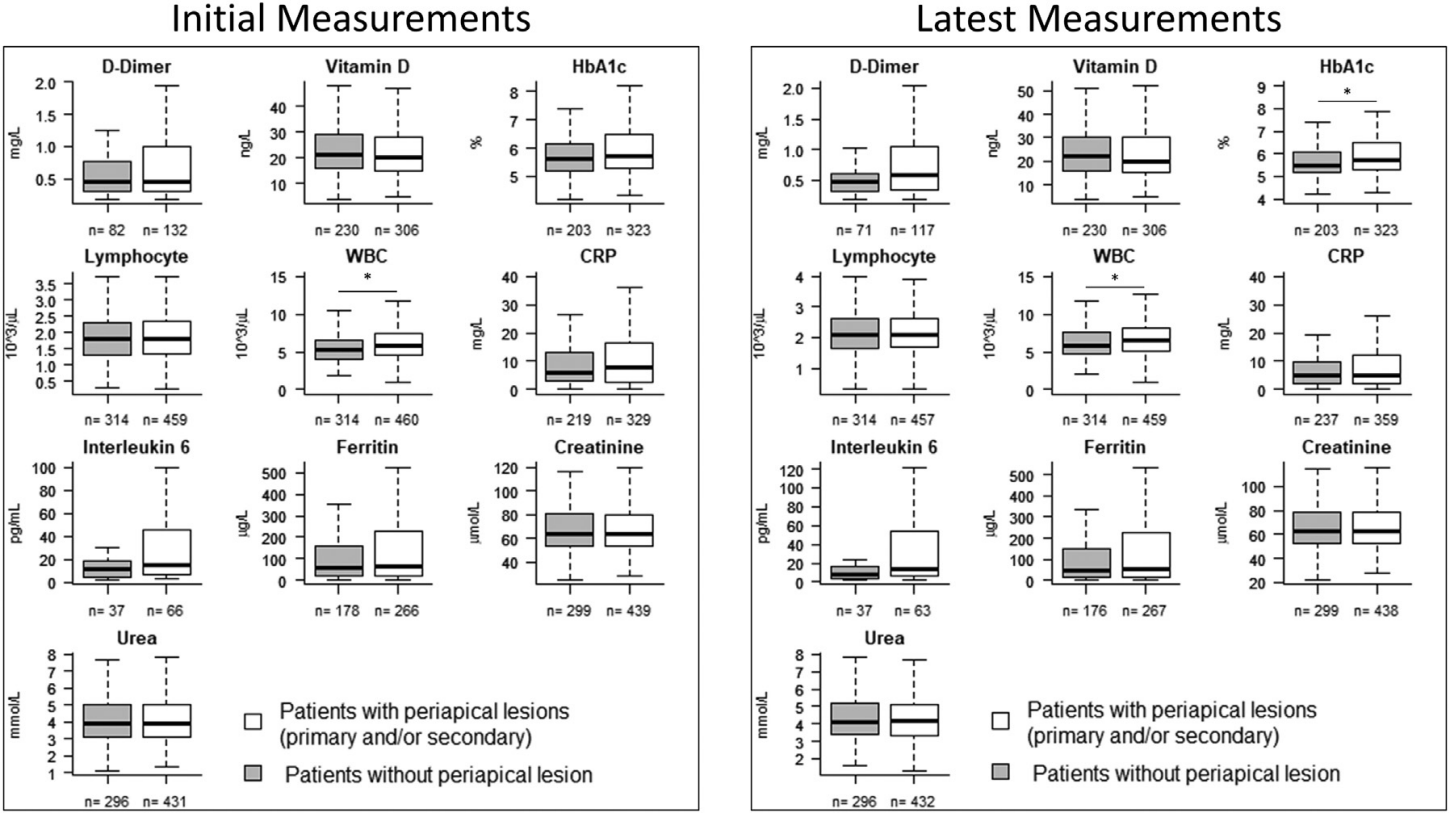
Box plots of the blood work of COVID-19 patients on admission and discharge comparing patients without periapical lesions with those with periapical lesions. The brackets indicate significant differences between groups (*P* < .05). n, number of patients analysed for each parameter and condition. Statistical analyses were done using the Mann–Whitney *U* test.

Amongst the 949 patients included, 63 faced COVID-19 complications (cases), whilst 886 did not (controls). Out of the 949 included patients, 552 had at least 1 AP lesion. Table 1 compares the demographic and medical characteristics of patients who had AP with those who did not. Risk analysis showed that patients with AP were significantly associated with male sex and diabetes. The presence of AP was not significantly associated with BMI, hypertension, asthma, cancer, chronic liver disease, chronic kidney disease, organ transplant, and immunocompromising conditions. Also, there were no significant differences in terms of average age between patients with AP (41.0 ± 13.8 years) and those without (41.3 ± 13.8 years) (*P* = .711), the number of dental appointments given, and cancellation of dental appointment between patients with AP and patients without AP. However, patients with AP attended significantly fewer dental appointments and did not show up to significantly more appointments. The comparison of blood laboratory parameters in patients with AP vs patients without AP are shown in Figure 1. The results showed significantly higher levels of WBC and HbA1c in patients with AP, both on patient admission and discharge.

**Table 1:** Description of the study cohort demographics and medical conditions according to presence or absence of AP.

Patient characteristics	No AP lesion (*n* = 397)	AP ≥1 lesion (*n* = 552)	*P* value
Age, y, mean (±SD)	41.3 (±13.8)	41.0 (±13.8)	.711
Female, No. (%)	236 (59.5%)	303 (54.9%)	.164
BMI >25, No. (%)	288 (72.5%)	410 (74.3%)	.748
Citizen, No. (%)	225 (56.7%)	282 (51.1%)	.099
Smoker, No. (%)	39 (9.8%)	82 (14.9%)	.023*
Hypertension, No. (%)	91 (22.9%)	128 (23.2%)	.938
Diabetes, No. (%)	113 (28.5%)	181 (32.8%)	.176
Asthma, No. (%)	55 (13.9%)	77 (13.9%)	1
Autoimmune, No. (%)	6 (1.5%)	7 (1.3%)	.783
Cardiovascular, No. (%)	81 (20.4%)	110 (19.9%)	.87
COPD, No. (%)	3 (0.8%)	4 (0.7%)	1
Cancer, No. (%)	9 (2.3%)	13 (2.4%)	1
Chronic kidney disease, No. (%)	9 (2.3%)	20 (3.6%)	.257
Chronic liver disease, No. (%)	5 (1.3%)	17 (3.1%)	.08*
Organ transplant, No. (%)	3 (0.8%)	8 (1.5%)	.375
Immunocompromised, No. (%)	9 (2.3%)	15 (2.7%)	.835
No. of dental appointments received, mean (±SD)	7.9 (±8.6)	6.9 (±7.3)	.131
No. of broken appointments, mean (±SD)	2.1 (±1.4)	2.4 (±2.0)	.019*
No. of appointments attended, mean (±SD)	6.7 (±7.9)	5.7 (±6.3)	.04*
No. of cancelled appointments, mean (±SD)	2.4 (±2.0)	2.4 (±1.9)	.637

⁎ Significant. Continuous variables were tested by independent *t* test (2-sided). Categorical variables were tested with Fisher exact test (2-sided). AP, periapical periodontitis (primary and/or secondary); BMI, body mass index; COPD, chronic obstructive pulmonary disease.

AP lesions were found in 49 of the 63 cases and in 503 of the 886 controls. Odds ratio analysis revealed significant association between AP and COVID-19 complications (OR, 2.67; 95% CI, 1.45–4.90). The logistic regression model used to calculate the odds ratio was then adjusted to medical and demographic factors known to be associated with COVID-19 outcomes and oral health. The factors in the logistic regression model included the following covariates: age, sex, periodontitis, citizenship, smoking, hypertension, BMI, diabetes, number of attended dental appointments per year, asthma, autoimmune diseases, cardiovascular diseases, chronic obstructive pulmonary diseases, cancer, chronic kidney diseases, chronic liver diseases, history of organ transplantation, and immunocompromising conditions. The OR remained significant after adjustment for comorbidities (adjusted OR [aOR], 2.98; 95% CI, 1.29–5.75; *P* = .008; Table 2). The presence of AP was also significantly associated with mechanical ventilation (aOR, 3.50; 95% CI, 1.07–11.9; *P* = .039) and ICU admission (aOR, 3.20; 95% CI, 1.45–7.25; *P* = .004), but not with death (Table 2).

**Table 2:** Logistic regression analysis for risk of COVID-19 complications in patients with AP adjusted to the presence of other comorbidities.

COVID-19 complications			OR (95% CI)	aOR (95% CI)*	*P* value
	No complication	Any complication			
No AP	383	14	1	1	
AP	503	49	2.67 (1.45–4.90)	2.98 (1.46–6.06)	.03
	No complication	Ventilation			
No AP	383	5	1	1	
AP	503	24	3.66 (1.38–9.67)	3.75 (1.23–11.49)	.02
	No complication	ICU admission			
No AP	383	11	1	1	
AP	503	46	3.18 (1.63–6.23)	3.58 (1.66–7.75)	.001
	No complication	Death			
No AP	383	5	1	1	
AP	503	11	1.68 (0.58–4.86)	2.43 (0.47–12.66)	.291

AP, periapical periodontitis (primary and/or secondary); OR, odds ratio; aOR, adjusted odds ratio; ICU, intensive care unit.

⁎ Adjusted for sex, age, periodontitis, citizenship, smoking, hypertension, body mass index, diabetes, and number of other comorbidities. Number of dental appointments seen as a health-awareness marker.

**Fig. 2:**
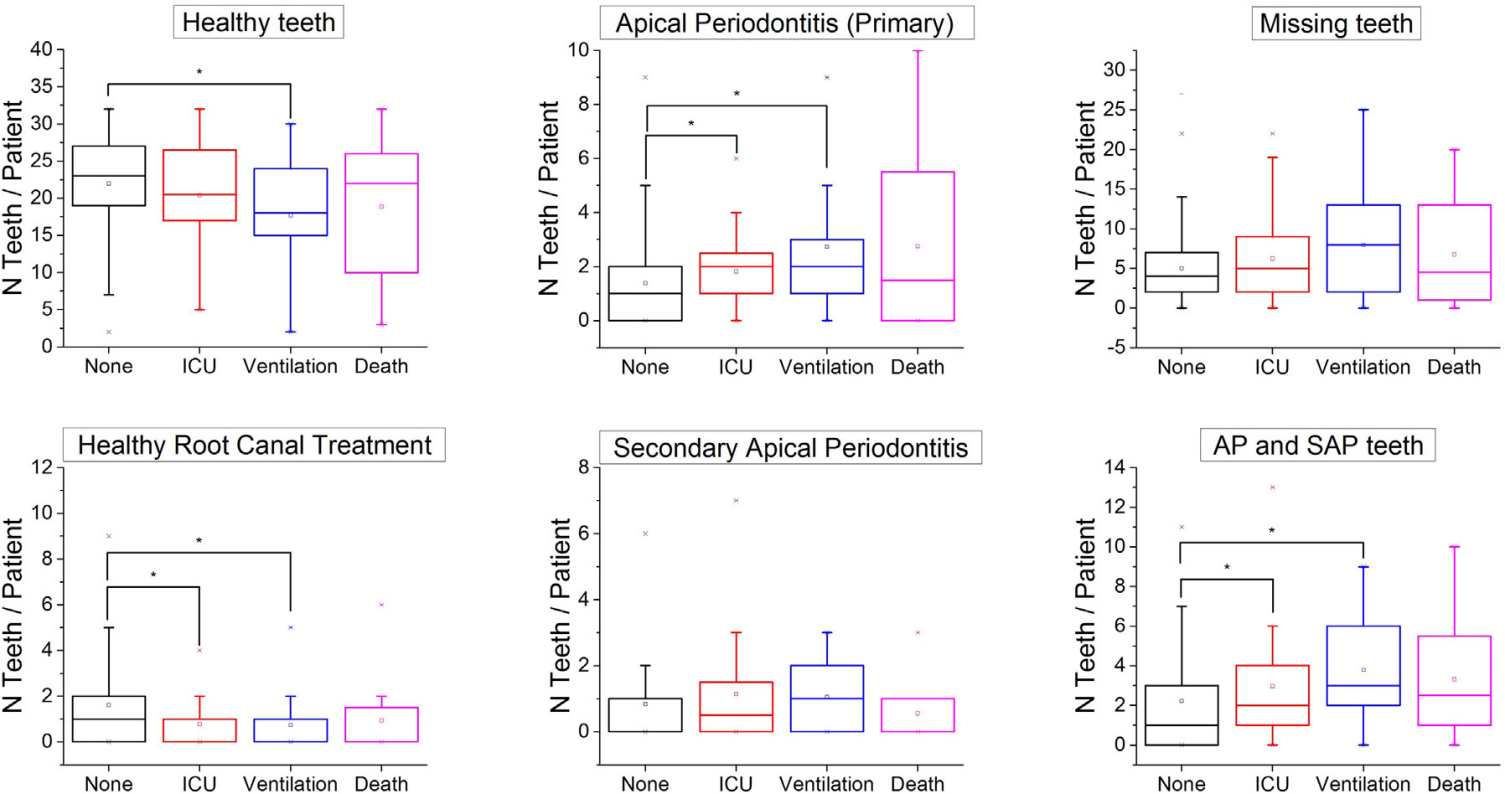
Box plots comparing COVID-19 patients without complications and those with complications in terms of number of teeth affected with various conditions: primary apical periodontitis (AP), secondary apical periodontitis (SAP), AP and SAP, healthy teeth, and teeth with healthy root canal treatments. *Significant difference (Kruskal–Wallis test) between groups (*P* < .05).

The included 949 OPGs taken before SARS-CoV-2 infection showed a total of 6565 (21.6%) missing/extracted teeth (X), 18,081 (59.5%) healthy teeth with no AP (H), 2216 (7.3%) teeth with primary apical periodontitis (PAP), 812 (2.7%) teeth with secondary apical lesion (SAP), 1484 (4.9%) teeth with HRCT, and 1210 (4.0%) teeth that could not be assessed (NA). Tooth-level analysis revealed a correlation between COVID-19 severity and the number of teeth with lesions (Figure 2). Patients with more severe COVID-19 complications tended to have fewer healthy teeth and more teeth with apical lesions than patients with milder complications or no complications. Patients who underwent ventilation had significantly fewer healthy teeth and fewer healthy RCTs and more teeth with AP than patients without COVID-19 complications did. Also, patients admitted to the ICU had significantly more teeth with AP and fewer teeth with healthy RCT than patients without COVID-19 complications. There was no significant difference in number of missing teeth and teeth with secondary AP regardless of COVID-19 severity. Regarding the associations between blood biomarkers and the number of periapical lesions, patients with more apical lesions had significantly higher initial levels of IL-6, WBC, and HbA1c and higher latest levels of WBC, CRP, and HbA1c (Supplementary Table 1). Many patients who presented initial HbA1c measurements of 6 or more did not have a diagnosis of diabetes in their records. This could indicate the presence of undiagnosed diabetes in our cohort. To address this issue, we investigated the association between AP and COVID-19 severity by stratifying our study population according to their HbA1c measurements. The results showed that AP patients had higher risk of COVID-19 complications regardless of HbA1c values (Supplementary Table 2).

Subgroup analysis according to the presence of AP and radiologic signs of periodontitis was conducted by stratifying patients with oral infections into 3 categories in order to assess the synergistic effect of AP and periodontitis: patients with AP but no periodontitis (only AP), patients with periodontitis but no AP (only periodontitis), and patients with both AP and periodontitis (Supplementary Table 3). These patients were compared to healthy patients without either periodontitis or AP. Risk analysis revealed that patients with both AP and periodontitis were at a higher risk for COVID-19 complications than those with only AP. Patients with both AP and periodontitis were significantly associated with higher risk of COVID-19 complications (aOR, 2.79; 95% CI, 1.04–7.52) and ICU admission (aOR, 3.25; 95% CI, 1.13–9.35) compared to healthy patients (Supplementary Table 3).

**Fig. 3:**
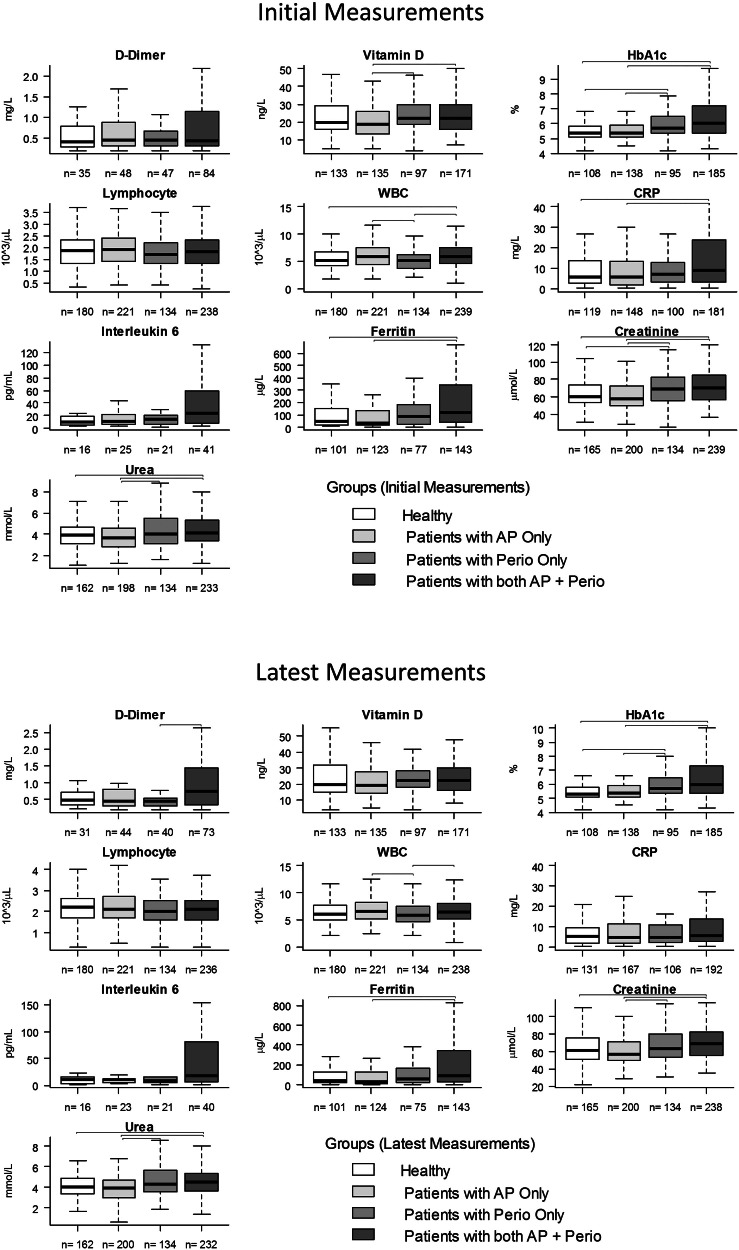
Box plots of the blood work of COVID-19 patients on admission and discharge comparing them according to the presence of apical periodontitis (AP) and periodontitis. Patients were divided into 4 groups: patients with both signs of AP and chronic periodontitis, patients with only signs of apical periodontitis, patients with only signs of chronic periodontitis, and patients without signs of AP or periodontitis (healthy). Brackets indicate significant differences between groups (*P* < .05). n, number of patients analysed for each parameter and condition. Statistical analyses were done using the Kruskal–Wallis test.

Initial blood analyses on COVID-19 diagnosis showed that patients with both AP and periodontitis had higher CRP, ferritin, urea, HbA1c, WBC, and creatine levels than healthy patients. Patients with both AP and periodontitis also had higher levels of WBC than periodontitis patients without AP (Figure 3). Periodontitis patients had higher levels of urea and vitamin D than AP patients and higher levels of creatine and HbA1c than healthy and AP patients. AP patients had higher levels of WBC than periodontitis patients.

Blood analyses on patient discharge showed that patients with both AP and periodontitis had higher levels of D-dimer than patients with only periodontitis, higher levels of HbA1C and ferritin than healthy patients and AP patients without periodontitis, and higher levels of creatine than patients with AP. Periodontitis patients had higher levels of HbA1C than healthy and AP patients and higher levels of creatine than AP patients. AP patients had higher levels of WBC than periodontitis patients.

## Discussion

This study found that the risk of COVID-19 complications was significantly higher amongst patients with AP compared to those without AP. In order to adjust for possible confounders, we performed multivariate logistic regression modelling with enough statistical power. After adjustment, AP still had strong and significant associations with COVID-19 complications (aOR, 2.72; 95% CI, 1.30–5.68), ICU admission (aOR, 3.21; 95% CI, 1.45–7.14), and need for assisted ventilation (aOR, 3.37; 95% CI, 1.03–10.99). These findings were further confirmed by tooth-level analyses showing that patients with COVID-19 complications had more teeth with AP and laboratory observations showing that patients with AP had an increased inflammatory burden. Subgroup analysis of the patients according to the presence of periodontitis and AP showed that patients with both AP and periodontitis were at a higher risk of COVID-19 complications than those with only one of the conditions.

Our findings are in line with other studies associating AP with systemic conditions such as cardiovascular disease^31^^,^^32^ and diabetes.^24^ These associations between AP and systemic health could be through the bacteraemia and increased systemic inflammatory burden caused by the chronic infection.^33^ In our study, we observed significantly increased levels of WBC in COVID-19 patients with AP, which is also a feature of severe COVID-19.^33^ Also, COVID-19 patients tended to have higher levels of CRP and IL-6. Thus, it could be speculated that the presence of AP undermines patients’ response to COVID-19 by creating an increased inflammatory burden.

It has also been proposed that the association between oral health conditions and COVID-19 severity could also derive from predisposing conditions common to both oral health and COVID-19, such as genetic variants and diabetes.^28^ Indeed, AP patients had significantly higher levels of HbA1c, which could highlight a link between AP and undiagnosed diabetes. Many AP patients who did not have a diagnosis of diabetes in their records presented initial HbA1c measurements of 6 or more. Even though these HbA1c measurements could be biased by the fact that they were taken after infection, they could still indicate that many patients in the cohort could have had undiagnosed diabetes, which might be linked to the presence of AP. To address this question, we investigated the association between AP and COVID-19 severity by stratifying our study population according to their HbA1c measurements. The results showed that AP patients had higher risk of COVID-19 complications regardless of HbA1c values.

Tooth-level analysis revealed a correlation between COVID-19 severity and the number of teeth with lesions (Figure 3). Patients with more severe COVID-19 complications tended to have fewer healthy teeth and more teeth with apical lesions than patients with milder complications or no complications whatsoever. Moreover, patients who underwent ventilation had significantly fewer healthy teeth and fewer healthy RCT and more teeth with AP than patients without COVID-19 complications. Also, patients admitted to the ICU had significantly more teeth with AP and fewer teeth with healthy RCT than patients without COVID-19 complications. There was no significant difference in number of missing teeth and teeth with secondary AP regardless of COVID-19 severity. Moreover, the number of teeth with periapical lesions was also correlated with the levels of blood biomarkers associated with COVID-19 severity such as WBC, HbA1c, and IL-6.

The association between AP and COVID-19 complications followed a gradient that further confirms our findings. Patients with more severe COVID-19 complications had more PAP, SAP, and AP lesions than patients without COVID-19 complications. This is in agreement with other studies reporting poorer overall oral health in patients with COVID-19 complications.^34^ Interestingly, there were no significant differences in the number of missing teeth as a function of COVID-19 complications. Missing teeth is a strong indicator of poor general health awareness; thus, the lack of significant difference would suggest that differences in healthy behaviour are probably not too large between patients with complications and those without.

An unexpected finding in our study was that patients with COVID-19 complications had fewer HRCTs than those without. This seems to indicate that patients with complications had more untreated AP lesions, whilst those without complications seem to have received appropriate root canal treatments. It has been previously reported that root canal treatments could have a protective association against cardiovascular disease mortality,^35^ but our results appear to suggest that it may also protect against COVID-19 complications. Future research on the benefits of root canal treatments on systemic health such as COVID-19 outcomes is warranted.

Stratified analysis revealed that patients with both AP and periodontitis were significantly associated with higher risk of COVID-19 complications (aOR, 2.79; 95% CI, 1.04–7.52) and ICU admission (aOR, 3.25; 95% CI, 1.13–9.35) compared to healthy patients. Patients who only had either AP or periodontitis were not significantly associated with COVID-19 complications. This could suggest that the association of AP with COVID-19 complications might not be specific, although our stratified analyses could be compromised by the relatively small sample size of the subgroups. The inflammatory burden observed in patients with AP was even higher when these patients also had signs of periodontitis. This seems to indicate that even though AP and periodontitis could independently influence systemic health, their combined presence could result in a more severe inflammatory burden. This is consistent with previously reported associations between dental damage and COVID-19 complications.^34^

One explanation for the association between the presence of AP lesions and COVID-19 severity could be the differences in health awareness. More health-aware patients could have better overall health, including better overall oral health, and therefore would be less likely to have complications. To address this potential confounding factor, we analysed indicators of oral health awareness such as the number of dental appointments attended by the patients in the last 3 years, and we adjusted our models accordingly. We observed that patients with AP attended significantly fewer dental appointments and missed more appointments. However, even after adjusting for these confounders, ORs associating AP with COVID-19 complications remained significant.

This study has several limitations, and thus our observation should be interpreted with caution. Given the study's observational retrospective design, our results cannot be interpreted as casual association between AP and COVID-19 severity. In other words, our findings do not necessarily indicate that AP causes severe COVID-19 complications. In addition, this retrospective study utilised electronic records of the health care system, which might be susceptible to gaps in relevant information from the clinical case that could lead to a potential bias. Also, even though our sample size was sufficiently large to assess our hypothesis, it was not large enough to explore various questions that arose in the course of our investigation, particularly, the specific role that important covariates, such as diabetes and periodontitis, could be playing in the associations between AP and COVID-19 complications. Studies with larger sample sizes are needed to address these points.

Another limitation of our study was that AP was only diagnosed radiologically using digital OPGs. Due to their relatively low radiation dose, OPGs are the radiographs of choice for general screening.^36^ However, even though digital OPGs have a better diagnostic value than conventional OPGs,^37^ they still have limited resolution compared to periapical radiographs and cone beam computed tomography (CBCT).^38^ Nonetheless, full-mouth series of periapical radiographs and CBCTs cannot be justified for screening due to ethical concerns. Still, OPGs have high specificity in detecting AP in both endodontically treated and untreated teeth, although their accuracy and sensitivity are limited.^39^^,^^40^ This means that our assessment could have underestimated the actual number of AP lesions. Indeed, there was an important number of teeth that could not be assessed properly (NA), especially amongst patients with severe COVID-19. To overcome these limitations, in our study, each radiograph was assessed by 2 calibrated, experienced endodontists, and disagreements were resolved by a third examiner. Calibrated experts with extensive clinical experience have shown higher interobserver agreement^10^ and reliability in detecting periapical pathology on OPGs.^41^

## Conclusions

Our case control study revealed that the presence of AP was associated with severe COVID-19 outcomes. COVID-19 patients with AP were associated with a significantly higher risk of complications (ie, ICU admission and need for mechanical ventilation) and presented higher blood levels of inflammatory and diabetes biomarkers (WBC and HbA1c) than those without apical periodontitis.

## Conflict of interest

None disclosed.

## Appendix Supplementary materials

Image, application 1

Image, application 2

## Footnotes

Supplementary material associated with this article can be found in the online version at doi:10.1016/j.identj.2024.01.002.

## References

[1] Rothan HA, Byrareddy SN. (2020). The epidemiology and pathogenesis of coronavirus disease (COVID-19) outbreak. J Autoimmun.

[2] Centers for Disease Control and Prevention. CDC updates, expands list of people at risk of severe COVID-19 illness. Available from: https://www.cdc.gov/media/releases/2020/p0625-update-expands-covid-19. Accessed 18 December 2021.

[3] Li Q, Ding X, Xia G (2020). Eosinopenia and elevated C-reactive protein facilitate triage of COVID-19 patients in fever clinic: a retrospective case-control study. EClinicalMedicine.

[4] Huang C, Wang Y, Li X (2020). Clinical features of patients infected with 2019 novel coronavirus in Wuhan, China. Lancet.

[5] Kamel AHM, Basuoni A, Salem ZA (2021). The impact of oral health status on COVID-19 severity, recovery period and C-reactive protein values. Br Dent J.

[6] Larvin H, Wilmott S, Kang J (2021). Additive effect of periodontal disease and obesity on COVID-19 outcomes. J Dent Res.

[7] Marouf N, Cai W, Said KN (2021). Association between periodontitis and severity of COVID-19 infection: a case–control study. J Clin Periodontol.

[8] Gupta S, Mohindra R, Singla M (2022). The clinical association between periodontitis and COVID-19. Clin Oral Investig.

[9] Tamimi F, Altigani S, Sanz M. (2022;). Periodontitis and coronavirus disease 2019. Periodontol 2000.

[10] Said KN, Al-Momani AM, Almaseeh JA (2022). Association of periodontal therapy, with inflammatory biomarkers and complications in COVID-19 patients: a case control study. Clin Oral Investig.

[11] Jakovljevic A, Nikolic N, Jacimovic J (2020). Prevalence of apical periodontitis and conventional nonsurgical root canal treatment in general adult population: an updated systematic review and meta-analysis of cross-sectional studies published between 2012 and 2020. J Endod.

[12] Boeddinghaus R, Whyte A. (2006). Dental panoramic tomography: an approach for the general radiologist. Australas Radiol.

[13] Chapman MN, Nadgir RN, Akman AS (2013). Periapical lucency around the tooth: radiologic evaluation and differential diagnosis. RadioGraphics.

[14] Dugas NN, Lawrence HP, Teplitsky PE (2003). Periapical health and treatment quality assessment of root-filled teeth in two Canadian populations. Int Endod J.

[15] Jiménez-Pinzón A, Segura-Egea JJ, Poyato-Ferrera M (2004). Prevalence of apical periodontitis and frequency of root-filled teeth in an adult Spanish population. Int Endod J.

[16] Tibúrcio-Machado CS, Michelon C, Zanatta FB (2021). The global prevalence of apical periodontitis: a systematic review and meta-analysis. Int Endod J.

[17] Gomes MS, Blattner TC, Sant'Ana Filho M (2013). Can apical periodontitis modify systemic levels of inflammatory markers? A systematic review and meta-analysis. J Endod.

[18] Gomes MS, Hugo FN, Hilgert JB (2016;). Apical periodontitis and incident cardiovascular events in the Baltimore Longitudinal Study of Ageing. Int Endod J.

[19] Georgiou AC, Crielaard W, Armenis I (2019). Apical periodontitis is associated with elevated concentrations of inflammatory mediators in peripheral blood: a systematic review and meta-analysis. J Endod.

[20] Georgiou AC, Cornejo Ulloa P, Van Kessel GMH (2021). Reactive oxygen species can be traced locally and systemically in apical periodontitis: a systematic review. Arch Oral Biol.

[21] Gomes MS, Blattner TC, Sant'Ana Filho M (2013). Can apical periodontitis modify systemic levels of inflammatory markers? A systematic review and meta-analysis. J Endod.

[22] Bae KS, Baumgartner JC, Shearer TR, David LL. (1997). Occurrence of *Prevotella nigrescens* and *Prevotella intermedia* in infections of endodontic origin. J Endod.

[23] Liljestrand JM, Mäntylä P, Paju S (2016). Association of endodontic lesions with coronary artery disease. J Dent Res.

[24] Nagendrababu V, Segura-Egea JJ, Fouad AF (2020). Association between diabetes and the outcome of root canal treatment in adults: an umbrella review. Int Endod J.

[25] Jansson L. (2015). Relationship between apical periodontitis and marginal bone loss at individual level from a general population. Int Dent J.

[26] Hajishengallis G. (2015). Periodontitis: from microbial immune subversion to systemic inflammation. Nat Rev Immunol.

[27] Altaie AM, Saddik B, Alsaegh MA (2021). Prevalence of unculturable bacteria in the periapical abscess: a systematic review and meta-analysis. PLoS One.

[28] Cai W, Marouf N, Said K. (2021). Nature of the interplay between periodontal diseases and COVID-19. Front Dent Med.

[29] Crocombe LA, Broadbent JM, Thomson WM (2012). Impact of dental visiting trajectory patterns on clinical oral health and oral health-related quality of life. J Public Health Dent.

[30] Orstavik D, Kerekes K, Eriksen HM. (1986). The periapical index: a scoring system for radiographic assessment of apical periodontitis. Dent Traumatol.

[31] Jakovljevic A, Duncan HF, Nagendrababu V (2020). Association between cardiovascular diseases and apical periodontitis: an umbrella review. Int Endod J.

[32] Caplan DJ, Chasen JB, Krall EA (2006). Lesions of endodontic origin and risk of coronary heart disease. J Dent Res.

[33] Sinha P, Matthay MA, Calfee CS. (2020). Is a “cytokine storm” relevant to COVID-19?. JAMA Intern Med.

[34] Sirin DA, Ozcelik F. (2021). The relationship between COVID-19 and the dental damage stage is determined by radiological examination. Oral Radiol.

[35] Virtanen E, Nurmi T, Söder P-Ö (2017). Apical periodontitis associates with cardiovascular diseases: a cross-sectional study from Sweden. BMC Oral Health.

[36] Bodey TE, Loushine RJ, West LA (2003). A retrospective study evaluating the use of the panoramic radiograph in endodontics. Mil Med.

[37] Sabarudin A, Tiau YJ. (2013). Image quality assessment in panoramic dental radiography: a comparative study between conventional and digital systems. Quant Imaging Med Surg.

[38] Estrela C, Bueno MR, Leles CR (2008). Accuracy of cone beam computed tomography and panoramic and periapical radiography for detection of apical periodontitis. J Endod.

[39] Nardi C, Calistri L, Pradella S (2017). Accuracy of orthopantomography for apical periodontitis without endodontic treatment. J Endod.

[40] Nardi C, Calistri L, Grazzini G (2018). Is panoramic radiography an accurate imaging technique for the detection of endodontically treated asymptomatic apical periodontitis?. J Endod.

[41] Sebring D, Kvist T, Buhlin K (2021). Calibration improves observer reliability in detecting periapical pathology on panoramic radiographs. Acta Odontol Scand.

