# Supplementary material for: COVID-19 Severity in Patients With Apical Periodontitis: A Case Control Study
Source: Int Dent J. 2024 Jan 20;74(4):736–45. doi: 10.1016/j.identj.2024.01.002 (PMC11287187; doi:10.1016/j.identj.2024.01.002)
Supplement: Supplementary file 1 [file mmc1.docx]

**Supplementary Table 1:** Association between blood biomarkers and number of teeth periapical lesion per patient

| Blood biomarker | Spearman Correlation Coefficient | p value | N |
| --- | --- | --- | --- |
| **Initial levels** |  |  |  |
| D-Dimer | .037 | .589 | 214 |
| Vitamin D | -.047 | .280 | 536 |
| HbA1c | .096* | .028* | 526 |
| Lymphocyte | .029 | .417 | 773 |
| WBCI | .107* | .003* | 774 |
| CRPI | .067 | .119 | 548 |
| Interleukin6 | .217* | .028* | 103 |
| Ferritin | .014 | .767 | 444 |
| Creatinine | .006 | .867 | 738 |
| **Latest levels** |  |  |  |
| D-Dimer | .076 | .303 | 188 |
| Vitamin D | -.054 | .208 | 536 |
| HbA1c | .100* | .022* | 526 |
| Lymphocyte | .009 | .795 | 771 |
| WBC | .078* | .031* | 773 |
| CRP | .082* | .045* | 596 |
| Interleukin-6 | .195 | .051 | 100 |
| Ferritin | .030 | .528 | 443 |
| Creatinine | .021 | .567 | 737 |
| Urea | -.032 | .395 | 728 |

*: denotes statistical significance

**Supplementary Table 2:** Association between AP and COVID-19 complications as a function of Hb1ca levels.

|  | No AP | AP | OR (95%CI) |
| --- | --- | --- | --- |
| Patients with Hb1ca < 6 | |  |  |
| No complications | 90 | 237 |  |
| Death | 0 | 0 | NA |
| ICU | 2 | 14 | 2.66(0.59-11.93) |
| Ventilation | 1 | 4 | 1.52(0.17-13.77) |
| Any complication | 2 | 14 | 2.66(0.59-11.93 |
| Patients with Hb1ca ≥6 | | |  |
| No complications | 37 | 115 |  |
| Death | 1 | 5 | 1.61(0.18-14.21) |
| ICU | 2 | 26 | 4.18(0.95-18.47) |
| Ventilation | 1 | 14 | 4.50(0.57-35.42) |
| Any complication | 3 | 28 | 3.00(0.86-10.45) |

**Supplementary Table 3:** Synergistic effect of periodontitis and AP: Sub analysis of risk of covid-19 complications associated with the presence of apical periodontitis and chronic periodontitis.

| Oral pathology present | COVID-19 Complication | | OR | AOR* | p |
| --- | --- | --- | --- | --- | --- |
|  | None | Any |  |  |  |
| Healthy | 226 (26.1%) | 9 (14.3%) | 1 | 1 |  |
| AP Only (without periodontitis) | 268(31.0%) | 12(19%) | 1.12(0.47-2.72) | 2.37(0.76-7.37) | 0.136 |
| AP and Periodontitis | 235(27.1%) | 37(58.7%) | **3.95(1.87-8.38)** | **2.79(1.04-7.52)** | **0.043** |
|  | None | Death |  |  |  |
| None | 226 (26.1%) | 4(25.0%) | 1 | 1 |  |
| Only AP (without periodontitis) | 268(31.0%) | 1(6.3%) | 0.21(0.023-1.9) | 1.63(0.07-41.67) | 0.766 |
| AP and Periodontitis | 235(27.1%) | 10(62.5%) | 2.40(0.74-7.78) | 3.62(0.36-35.71) | 0.273 |
|  | None | ICU |  |  |  |
| None | 226 (26.1%) | 7(12.3%) |  |  |  |
| Only AP (without periodontitis) | 268(31.0%) | 12(21.1%) | 1.45(0.56-3.73) | 2.87(0.87-9.52) | 0.085 |
| AP and Periodontitis | 235(27.1%) | 34(59.6%) | **4.67(2.03-10.75)** | **3.25(1.13-9.35)** | **0.028** |
|  | None | Ventilation |  |  |  |
| None | 226 (26.1%) | 2(6.9%) | 1 | 1 |  |
| Only AP (without periodontitis) | 268(31.0%) | 4(13.8%) | 1.69(0.31-9.29) | 4.99(0.48-5.26) | 0.179 |
| AP and Periodontitis | 235(27.1%) | 20(69.0%) | 9.62(2.22-41.62) | 6.54(0.77-5.56) | 0.085 |

Healthy: patients with neither periodontitis nor AP. Only Periodontitis: patients with periodontitis but no AP. Only AP: patients with AP but no signs of periodontitis. AP and Periodontitis: patients with both AP and periodontitis. OR: Odds ratio. AOR: adjusted odds ratio. * model adjusted to sex, age, citizenship, smoking, hypertension, BMI, diabetes, and number of other comorbidities.
